# Distribution of household disinfection kits during the 2014-2015 Ebola virus outbreak in Monrovia, Liberia: The MSF experience

**DOI:** 10.1371/journal.pntd.0008539

**Published:** 2020-09-21

**Authors:** Engy Ali, Guido Benedetti, Rafael Van den Bergh, Anna Halford, Luke Bawo, Moses Massaquoi, Saverio Bellizzi, Peter Maes

**Affiliations:** 1 Médecins Sans Frontières–Operational Centre Brussels, Medical Department, Luxembourg Operational Research Unit (LuxOR), Luxembourg, Luxembourg; 2 Médecins Sans Frontières–Operational Centre Brussels, Monrovia, Liberia; 3 Ministry of Health and Social Welfare, Monrovia, Liberia; 4 Médecins Sans Frontières–Operational Centre Brussels, Medical Department, Brussels, Belgium; Center for Disease Control and Prevention, UNITED STATES

## Abstract

During the initial phase of the 2014–2016 Ebola virus disease (EVD) outbreak in Monrovia, Liberia, all hospitals’ isolation capacities were overwhelmed by the sheer caseload. As a stop-gap measure to halt transmission, Medecins sans Frontieres (MSF) distributed household disinfection kits to those who were at high risk of EVD contamination. The kit contained chlorine and personal protective materials to be used for the care of a sick person or the handling of a dead body. This intervention was novel and controversial for MSF. This paper shed the light on this experience of distribution in Monrovia and assess if kits were properly used by recipients. Targeted distribution was conducted to those at high risk of EVD (relatives of confirmed EVD cases) and health staff. Mass distributions were also conducted to households in the most EVD affected urban districts. A health promotion strategy focused on the purpose and use of the kit was integrated into the distribution. Follow-up phone calls to recipients were conducted to enquire about the use of the kit. Overall, 65,609 kits were distributed between September and November 2014. A total of 1,386 recipients were reached by phone. A total of 60 cases of sickness and/or death occurred in households who received a kit. The majority of these (46, 10%) were in households of relatives of confirmed EVD cases. Overall, usage of the kits was documented in 56 out of 60 affected households. Out of the 1322 households that did not experience sickness and/or death after the distribution, 583 (44%) made use of elements of the kit, mainly (94%) chlorine for hand-washing. At the peak of an EVD outbreak, the distribution of household disinfection kits was feasible and kits were appropriately used by the majority of recipients. In similar circumstances in the future, the intervention should be considered.

## Author summary

Liberia was one of the worst countries hit by the 2014–2016 Ebola Virus outbreak (EVD). All hospitals’ isolation capacity in Monrovia was stretched by the spiralling number of EVD cases. As a stop-gap measure, Medecins Sans Frontieres (MSF) distributed household disinfection kit to those who were considered at high risk of EVD transmission, including relatives of confirmed EVD cases, health staff and households in the most affected districts across Monrovia. The purpose of the kit was to care for sick person while waiting for an ambulance or handling dead body while waiting for burial team. The kit contained chlorine and personal protective materials. Health promotion strategy on the purpose and use of the kit was integrated into the distribution. Follow up with the kit recipients was done by phone to better understand the use of the kit. Overall, 65,609 kits were distributed between September and November 2014. Among 1,386 recipients reached by phone, 60 cases of sickness and/or death events occurred in households which received a kit. The majority of these were among the relatives of confirmed EVD cases. Kits’ use was documented in 56 out of 60 affected households. The distribution of household disinfection kits was feasible and kits were appropriately used by the majority of the recipients. In similar circumstances in future, the intervention should be considered.

## Introduction

The West Africa Ebola Virus Disease (EVD) outbreak in 2014–2016 was the first and so far only EVD outbreak to affect several major urban areas. Liberia was one of the worst affected countries, with the capital Monrovia seeing a rapid spiralling of EVD cases from August 2014 onwards. By the end of the outbreak in Liberia, in June 2016, 10,678 EVD cases had been declared, and 4810 deaths had occurred [1,2].

Médecins Sans Frontières (MSF) was one of the first international organisations responding to the EVD outbreak in the overcrowded urban context of Monrovia. In general, the MSF outbreak management strategy incorporates case isolation, contact tracing, outreach activities, safe burials, health promotion, and psycho-social support [3], though MSF does not necessarily perform each of these activities itself. In the initial phase of the outbreak in Monrovia (August 2014), the health system failed as all hospitals’ isolation capacities were overwhelmed by the sheer caseload. Even while MSF was setting up its largest-ever Ebola Treatment Centre (ETC), ELWA3 (with a final capacity of 240 beds), it found itself in the unprecedented situation of having to send ill patients home, as there was nowhere they could be isolated [4,5]. Clearly, conventional measures of outbreak management were not capable of containing the epidemic in the urban context of Monrovia.

In the face of the overwhelmed isolation capacity in Monrovia, a stop-gap measure was launched by MSF to safeguard households where EVD cases were being cared for, and to attempt to curb the spread of the outbreak. The plan was to distribute household disinfection kits to relatives of confirmed EVD cases, and to others considered at high risk of contamination: health workers, and households in the most EVD-affected zones of the city. The kit contained chlorine and personal protective materials (bucket, surgical gown, mask and gloves) to be used for the care of a sick person while waiting for an ambulance, or the handling of a dead body while waiting for a burial team. The distribution was accompanied by several health promotion (HP) activities, in order to ensure the optimal and correct use of the kits. Follow-up phone calls to recipients were conducted, to reinforce the HP messages and to enquire about the use of the kit.

Evidence on similar strategies is limited, and is not related to EVD. In a systematic review on the impact of water, hygiene and sanitation interventions of cholera control, only one study addressed the distribution of household disinfection kits as a community-based preventive method during a large-scale cholera outbreak. which proved to be feasible, and valued by the target population [6].

This intervention in an urban EVD outbreak was novel and controversial for MSF: concerns were raised on the risk of disrupting the “no-touch” policy during mass distributions, the possible incorrect use of kits by the population and the challenge in processing potentially contaminated waste. We therefore set out to document the experience of the distribution in Monrovia and to assess if kits were properly used by recipients. The objectives of this study were to i) describe the distribution process of disinfection kits from September to November 2014 in Monrovia; and ii) describe the reported use of the kits by the recipients.

## Methods

### Study design

This was a descriptive study using operational monitoring data from the MSF response to the 2014–2015 EVD outbreak in Monrovia, Liberia. The study design and protocol is in item S1 Protocol.

### Setting

Monrovia is the capital and the most populous city (1.2 million inhabitants) in Liberia, accounting for 29% of the total population. It is located in Montserrado County and divided into administrative districts, which are subdivided into communities. After decades of civil war, the country has some of the worst health indicators in the world. In 2014, the estimated under-five mortality rate was 73 per 1,000 live births [7], while the per capita public expenditure on health was 13 US dollars and the health workforce density less than 3.7/10,000 population [8,9].

### The EVD outbreak in Liberia

Although the first EVD case was reported in West Africa on March 23^rd^, 2014, few cases were detected in Liberia till June 2014. By July 2014, the epidemic had reached Monrovia, and the President of Liberia declared a state of emergency on August 6^th^. By mid-August, the World Health Organization (WHO) estimated that 1,000 isolation beds were needed for EVD cases, capacity that remained largely unmet. Taxis reported transporting sick persons and their relatives/care-takers all over the city in the search of a hospital bed. Even MSF’s isolation capacity was overstretched, and patients had to be turned away from the 23^rd^ of August till the 20^th^ of September. A decline in the number of EVD cases was only observed in November 2014. The country was finally declared Ebola-free on June 9^th^, 2016 [10,11].

### Household disinfection kit distribution

The contents of the kit changed over the period of distribution; according to the needs of the population and the availability of items (e.g. goggles and taps to be inserted into the plastic buckets were added at a later stage). Box 1 describes the final contents of the kit. Preparation of the kits required significant logistical support from MSF and under time constrains (e.g. to affix taps to about 65,000 buckets).

**Box 1. Contents of the household disinfection kit distributed by Médecins Sans Frontières in response to the Ebola Virus Disease outbreak in Monrovia, Liberia, 2014**

Item (Units)

20.5 kilogram of disinfectant, chlorine powder (1)

Dosing spoon for chlorine powder (1)

Disposable latex gloves (100)

Bars of soap, 200 grams each (5)

Reusable rubber gloves for cleaning (4)

Plastic bucket with lid, 20 litres (1)

Plastic bucket with tap, 20 litres (1)

Plastic hand sprayer, household type (1)

Roll of plastic bags, 100 litres (20)

Basic goggles (1)

Disposable surgical masks (25)

Surgical vests, gown (4)

Leaflet explaining how to use the kit (1)

Two parallel distribution strategies were put in place, in agreement with the Ministry of Health (MoH). Targeted distribution was conducted to individuals at highest risk of exposure, and in a following phase, mass distributions were conducted to households in the most affected urban districts (mass distribution). The beneficiaries of the kits are described in Box 2. The targeted distribution to health staff was implemented by the MoH at facility level. The distribution to other at-risk population (non-health staff, and mass distribution) was conducted by MSF. Targeted distribution was implemented at ETCs or at household level.

**Box 2. Beneficiaries of the household disinfection kit distribution conducted by Médecins Sans Frontières in response to the Ebola Virus Disease outbreak in Monrovia, Liberia, 2014**

Targeted distribution

- Suspect cases turned away from the Médecins Sans Frontières (MSF) Ebola Treatment Centre (ETC) ELWA33 during the Ebola Virus Disease (EVD) peak period (23^rd^ August– 20^th^ September, 2014) due to insufficient isolation capacity
- Relatives/caretakers of EVD-confirmed patients admitted in the MSF ETC and other treatment units
- Health staff, medical and non-medical (MSF, MoH, Red Cross burial team members, ambulance drivers, other partner organizations)

Mass distribution

Households from four districts in Monrovia i.e. Clara Town, New Kru Town, Logan town and West Point (as the most populous, poor and heavily EVD-affected areas)

The mass distributions in the community were planned carefully to avoid any security incidents or disruption. The distribution aimed for one kit per household; the number of households per district and community were identified through the national census and verified with the community leaders. Specific measures to organize the distribution and avoid disruption or security incidents were put in place: i) community leaders were informed about the distribution and engaged in identifying the distribution sites in the community; ii) tokens were handed out by the community leaders 2–3 days before the distribution: each household received one token, to be exchanged for one kit at the distribution site; iii) HP sessions were conducted using live demonstrations (25 minutes, for 30 participants maximum) and movie projections (45 minutes, for up to 150 participants) 2–4 days before the distribution at all sites; iv) only women were allowed to pick up the kits at the distribution site, as it was easier for women to participate at an “women only” event in the early hours; v) community leaders were told about the exact date and time of distribution at 3.00 am on the actual day of distribution; and the distribution started at around 6:00 am. This measure was specifically designed to minimize overcrowding at the distribution sites; and vi) arrangements were made with local authorities in order to suspend the curfew (part of the national emergency measures) in certain areas.

The mass distributions were conducted by four MSF experienced teams, who had previous experience in mass distribution in other contexts. The teams followed a “mobile approach” through trucks and remained on site for a maximum of two hours: the perimeter of the site was fenced, the distribution occurred from a truck, and an evacuation route was always identified upfront in case of security incidents. The entire process was organized respecting a “no-touch” policy, and was on stand-by to be cancelled at any sign of unrest.

The HP strategy was integrated into the kit distribution: 8–15 health promoters participated in every distribution event. HP sessions and demonstrations focused on the purpose of the kit and how to use its contents. They were held in the areas of distribution, including the community and hospitals. HP messages emphasized that the kit should be only be used for either care of sick persons who were not able to be transferred to the ETC due to lack of isolation beds or limited transportation, or to be used during handling a dead body while waiting for the burial team. It was not expected that the kit would be used for any kind of disinfection purposes.

### Phone follow-up sessions

As the kit distribution was a new intervention and required enhanced monitoring, follow-up phone calls were conducted among recipients by four trained health promoters, between September 20^th^ and October 27^th^, 2014. Beneficiaries of the targeted distribution including relatives of confirmed cases admitted to ETC and health staff were requested to leave a phone number for follow-up. For the mass distribution, a random sample of recipients (roughly 2%) was identified at the time of distribution, and their phone numbers were also requested. Calls were planned for the week following the distribution, to inquire about the utilization of kits and to reinforce HP messages. A structured questionnaire (S1 Questionnaire) was used to gather information on the following: basic demographics of the recipient’s household, occurrence of illness or death in the recipient’s household after receiving the kit, and to assess the correct use of the different items.

The correct use of the different kit items was considered as follows: a) use of chlorine preparation for hand wash (0.05%) and cleaning surfaces (0.5%), hand sprayer for spraying 0.05% chlorine on contaminated items with body fluids before manipulation, b) use of gloves (thin & thick), face masks and surgical gowns when manipulating sick and dead persons or their body fluids, c) waste bags and instructions on disposal either by burning or burying.

The questionnaire was pre-tested by health promoters and amended accordingly. The purpose of the phone call was explained to all participants and verbal consent was obtained before administering the questionnaire.

### Data and analysis

Information on the distribution process was sourced from MSF operational reports, log books, and operational monitoring sheets. Results from phone interviews were single-entered by an encoder into a structured electronic spreadsheet (Excel 2010, Microsoft Corp, Redmond, WA, USA). Households reached by phone were categorised as beneficiaries of: i) targeted distribution for non-health staff; ii) targeted distribution for health staff; iii) mass distribution. A distinction was made between non-health and health staff on the assumption of different levels of health literacy at household level. Phone numbers of kits recipients were only collected from the 20^th^ of September, after the peak period of turning away suspects cases from the MSF ETC. Therefore this group were not contacted and not included in this analysis. Associations between experiencing sickness and/or death in the household after the distribution (yes/no) and the use of the kit (yes/no) were measured by Fisher’s exact test. Odds ratios (ORs) and 95% CIs were calculated by logistic regression to measure the strength of association among the following: i) beneficiaries, the experience of sickness and/or death after the distribution (exposures) and the use of the kit (outcome); ii) beneficiaries, the demographic profile of households (exposures) and the experience of sickness and/or death after the distribution (outcome). Response data are available in S1 Spreadsheet Analysis was done with STATA/IC Version 15.0. (StatCorp, College Station, TX, USA).

### Ethics

The study fulfilled the exemption criteria set by the Ethics Review Board (ERB) of MSF (Geneva, Switzerland) for a-posteriori analyses of routinely collected data. It was conducted with permission from the Medical Director of MSF Operational Centre Brussels, Belgium. The MSF response strategy and operations in Liberia were formally approved by the Ministry of Health and Social Welfare.

## Results

### Kit distribution

On the 7th of August 2014, MSF-Operational Centre Brussels decided to engage in the kit distribution. The implementation was delayed due to discussions on the potential adverse consequences of mass distribution in the community delayed the implementation, and particularly due to the enforced quarantine imposed by the government in the areas targeted for the distribution; the quarantine was expected to result in turmoil.

Three shipments of materials were organized; kits were imported to avoid interfering with the local economy and risks to pull out available disinfecting materials from the local market in Liberia. Most airlines refused to fly into Monrovia; only one agreed and under the condition that nobody approached the aircraft once it was on the ground, and the kits had to be ejected out of the plane. The population of beneficiaries for distribution was estimated at about 65,000 households in Monrovia. In the end, materials for 73,181 kits were shipped to Monrovia.

Mass distribution in the community was initially intended to target hotspots of transmission based on the occurrence of new cases. However, this was not feasible due to the poor surveillance system and high number of identifiable hotspots. Therefore, priority areas were identified based on attack rates at district level, and contextual factors which might favour the spread of the EVD (e.g. overcrowded slums). Mass distribution was carried out in the districts of New Kru Town, Clara Town and West Point. West Point hosted an informal settlement of around 34,600 inhabitants in 2015 [12]. Logan Town district was geographically between the other districts and was also included in the distribution. Unfortunately, a dedicated strategy for more than 9000 taxi and moto-taxis drivers in Monrovia, who were at high risk themselves from transporting patients with EVD disease as well as transmitting it to others, was logistically not feasible to implement.

**Fig 1:**
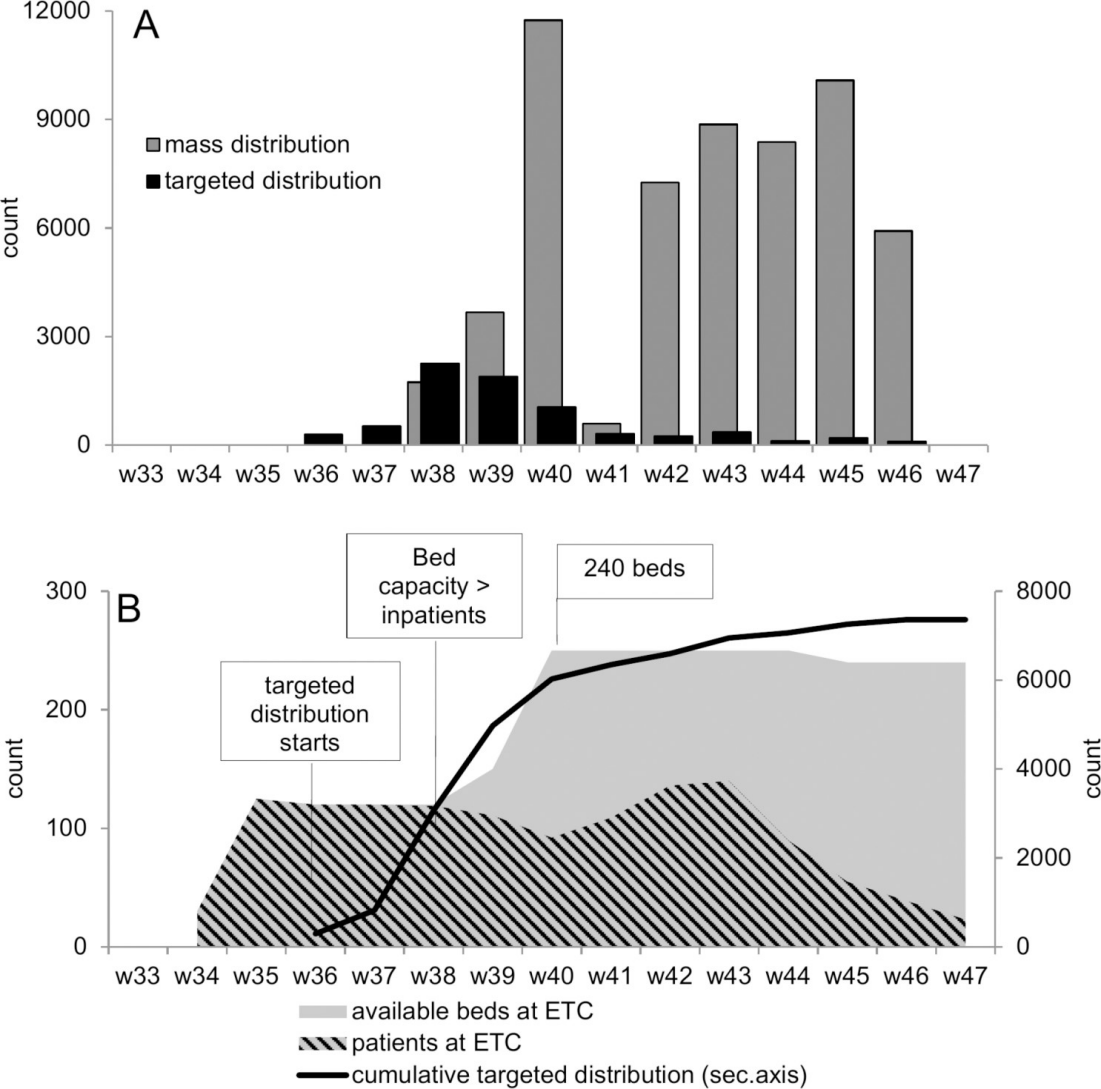
Household disinfection kit distributions by Médecins Sans Frontières in response to the Ebola Virus Disease outbreak in Monrovia, Liberia, 2014: A) number of distributed kits (mass and targeted distribution) per epidemiological week and B) cumulative number of distributed kits (targeted distribution), number of admitted patients and available isolation beds at the Médecins Sans Frontières Ebola Treatment Centre ELWA3.

Overall, 65,609 kits were distributed: 7,359 through targeted distribution and 58,250 through mass distribution. Fig 1 shows the roll-out of the distribution, starting in week 36 of 2014 for the high-risk beneficiaries (targeted distribution) when the isolation capacity of ETCs in Monrovia was overstretched. Distribution to health staff started in week 37. More than 800 kits were distributed through targeted distribution during the first two weeks. Overall, distribution was carried out through 91 distribution events, with a median of 50 kits distributed at each event [IQR 24–100]. Mass distribution was pre-tested in week 38 in the immediate vicinity of the MSF ETC, and was then scaled up into the chosen districts. In total, 67 mass distribution sessions took place, with a median of 649 [IQR 344–838] kits distributed/event/team. Notably in West Point, MSF distributed 13,633 kits (covering more than 99% of households) in five days. Activities were interrupted in week 41, as the distribution outpaced the manual assembly of the kits. On two occasions the team interrupted a specific distribution event, due to security issues, as per protocol.

### Kit utilization

**Fig 2:**
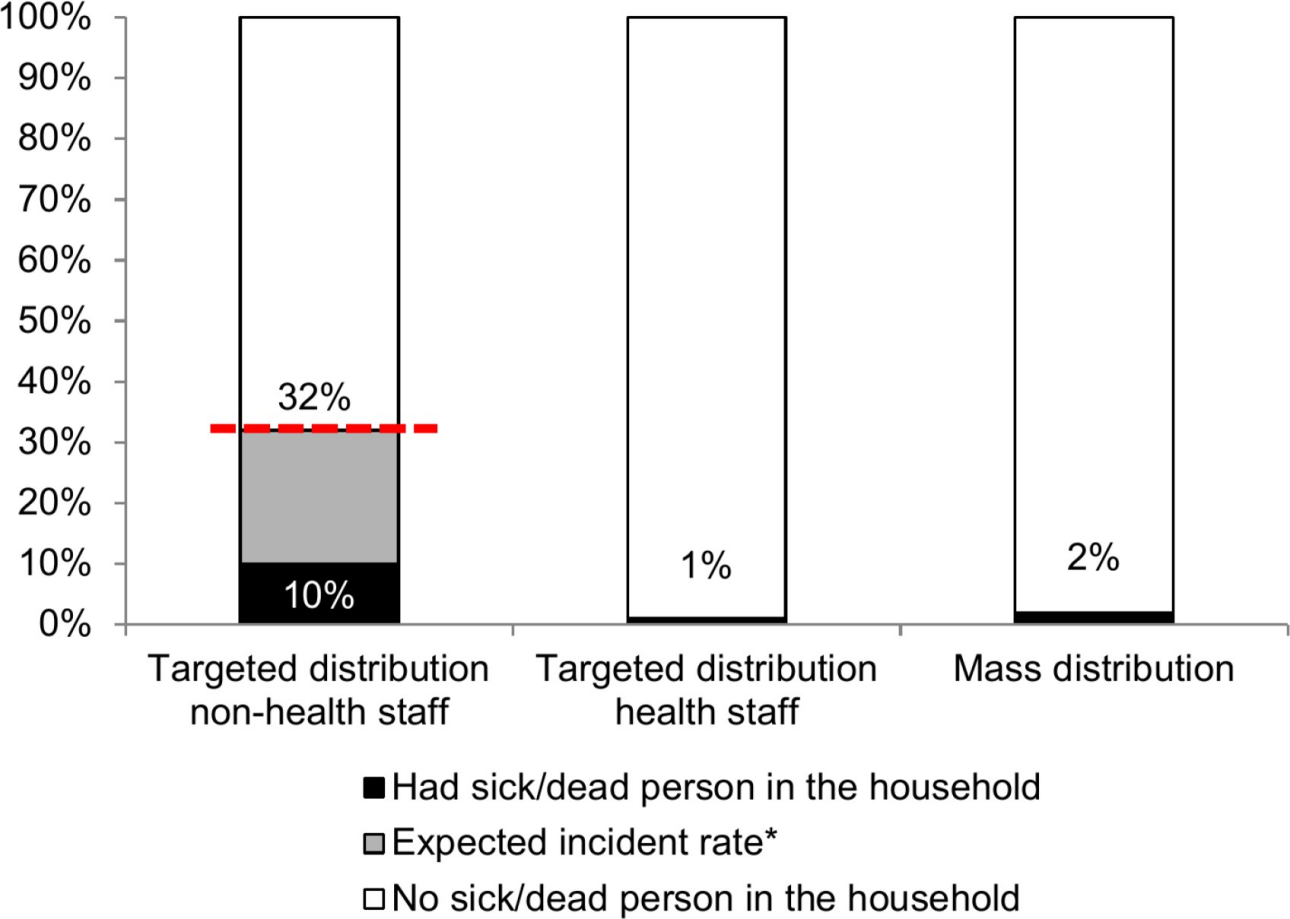
Incidence of sickness and/or death among household disinfection kit recipients, during the Médecins Sans Frontières response to the Ebola Virus Disease outbreak in Monrovia, Liberia, 2014. *Attack rate estimated at 32% for close household contacts [95% confidence interval (CI) 26–38%], without using the protective kit (Brainard et al, 2015).

A total of 1,386 recipients were reached by phone (Table 1), at a median time of five days [IQR 4–9] after receiving the kit. Households from the different groups had similar compositions. Fig 2 shows that after receiving the kit, households at the highest risk of EVD (targeted distribution for non-health staff) experienced more events of sickness and/or death (46, 10%) than the other groups (see supporting data in S1 Spreadsheet). The latter is compared in the graph to an expected attack rate of 32% reported by Brainard et al, 2015, for household contacts who have directly touched an EVD case [95% confidence interval (CI) 26–38%] and did not use the protective kit [13].

**Table 1:** Characteristics of household disinfection kit recipients followed up by phone, during the Médecins Sans Frontières response to the Ebola Virus Disease outbreak in Monrovia, Liberia, 2014. Response data are available in S1 Spreadsheet.

	Targeted distribution non-health staff	Targeted distribution health staff	Mass distribution
Total number of recipients reached by phone (%)	460 (33)	448 (32)	480 (35)
Days between distribution and phone FU, median [IQR]	8 [5–14]	4 [4–9]	5 [4–7]
People usually living in household, median [IQR]	7 [4–10]	7 [5–10]	6 [4–9]
People living in household at the time of FU, median [IQR]	6 [4–9]	7 [5–10]	6 [4–9]
Families living in household, median [IQR]	1 [1–1]	1 [1–1]	1 [1–2]

FU: Follow Up; IQR: inter-quartile range

Across the three groups, a total of 60 cases of sickness and/or death occurred in households who received a kit. Among these “affected” households, kit usage was assessed. No differences in usage rates of the kits were observed between the different recipients or between different household compositions. Overall, usage of the kits was documented in 56 out of 60 affected households (Table 2). All of them used chlorine for handwashing and cleaning purposes. The correct use of chlorine was reported by 87% (95%CI 75–94) for handwashing and 71% (95%CI 56–82) for cleaning. Overall, 94% reported the use of disposable gloves during manipulation of a sick or deceased person, and 81% used thick gloves to manipulate/clean possibly contaminated body fluids and soiled items. In terms of waste management, 93% (95%CI 80–98) followed the instructions and reported burning waste bags. Other disposal procedures were less well-performed before disposal: 79% disinfected the outside of a plastic bag and 73% placed a waste bag into another (Table 2). See supporting data in S1 Spreadsheet.

**Table 2:** Reported use of household disinfection kit items among households experiencing sickness and/or death, during the Médecins Sans Frontières response to the Ebola Virus Disease outbreak in Monrovia, Liberia, 2014.

	# out of total answers available	Percentage (95% confidence interval)
**Use of the kit**		
Using any item of kit	56 out of 59	95 (85–98)
Using chlorine for handwashing	56 out of 56	100
Correct* usage	47 out of 54	87 (75–94)
Using chlorine for cleaning purposes	53 out of 53	100
Correct** usage	36 out of 51	71 (56–82)
Using disposable gloves	46 out of 49	94 (82–98)
Washing gloves with chlorine between and after each contact	37 out of 48	77 (63–87)
Using thick gloves	38 out of 47	81 (67–90)
Using gown	39 out of 48	81 (67–90)
Using mask	40 out of 47	85 (71–93)
Spraying items of a sick person	36 out of 45	80 (65–90)
Using the hand sprayer for disinfection	44 out of 48	92 (79–97)
**Waste management**		
Disposing gloves after use	45 out of 48	94 (82–98)
Disinfecting the outside of plastic bag before disposal	38 out of 48	79 (65–89)
Placing a waste bag into another for disposal	35 out of 48	73 (58–84)
Burning waste bags	40 out of 43	93 (80–98)
Running out of kit items	14 out of 46	30 (19–46)
Reporting the kit to be useful	33 out of 33	100

*reported number of spoons per 20 litres

** reported number of spoons per bucket

Out of the 1322 households that did not experience sickness and/or death after the distribution, 583 (44%) made use of elements of the kit: 549 (94%) used the chlorine for hand-washing purposes and 361 (62%) for cleaning purposes. Correct use of chlorine was 87% and 71% for these purposes, respectively.

## Discussion

This the first study to documents the experience of distributing household disinfection kits during the unprecedented and overwhelming peak of the West Africa 2014–2016 EVD outbreak in Monrovia, Liberia. The distribution strategy proved to be feasible, despite the anticipated difficulties. The distribution process was implemented in a phased manner. Overall, there was a good uptake of the kit materials among all recipients, primarily among those who had a sick and/or dead person in their household, confirming the perceived relevance of the kit distribution and its accompanying health promotion activities. The correct use of kit items was encouraging for most items, though a number of instructions were followed less appropriately.

A number of operational findings and lessons learnt merit discussion. First, the intervention sparked considerable debate in MSF on whether the possible benefits outweighed the possible risks–these discussions likely delayed the intervention to some extent. The risks were focused around creating a false sense of security among recipients so they might not seek care; the distribution itself might result in additional EVD transmission among the population, primarily related to risk of disruption during distribution; the risk of handling contaminated waste materials; and population’s acceptance of MSF’s message to only use the kit in case of having sick or dead person in the household, while the dominant message in Monrovia was to use chlorine for disinfection purposes.

Despite the controversy and these anticipated risks, the strategy seemed to be relevant as an attempt to contain EVD transmission at household level, particularly during a crisis situation with a lack of isolation capacity. It should be emphasised that the kit was not designed to allow provision of home-based care, but rather to offer some protection, however imperfect, in case of sickness and/or death of a family member in a setting where immediate isolation could not be ensured. Despite these concerns, the purpose of the distribution seemed to be well-understood by the population, and no major incidents occurred. No analysis was done on whether the kits sparked a false sense of security, and this may require further investigation in case of future outbreaks.

Second, HP efforts played a key role in emphasising that kits should be used only for care of sick persons or in case of death while waiting for the ambulance or burial team. As a bonus, the kit distribution provided an opportunity for MSF to reinforce HP strategies related to EVD in the most unserved areas of Monrovia.

Third, replicability of this intervention in future outbreaks may be not be feasible, as not all actors have the logistical and human resources at their disposal for mounting this fairly complex strategy. In the end, MSF was the sole actor distributing kits during the peak of the EVD outbreak in Monrovia. Other actors suggested a similar measure to only distribute chlorine kits to provide households with appropriate means of disinfection but this did not happen.

It is currently unknown whether a positive effect in halting EVD transmission can be attributed to the kits. However, there is some evidence for the effectiveness of the intervention. Modelling studies suggest that the protective kits would reduce transmission under scenarios of exceeded ETC capacity, corresponding to a decrease in EVD transmission from 10% to 50% [14], suggesting that while these protective kits can complement the improvement in ETC capacity for case isolation to halt EVD transmission.

Moreover, our study showed that 10% of households at the highest risk of EVD (targeted distribution for non-health staff) had experienced sickness and/or death in the household, as compared to 32% among households without an intervention and reporting direct contact with a case, in a published meta-analysis. Another study showed EVD risk ranging from 83% for touching a dead body to 8% for minimal contact [15].

This study had a number of limitations: there were challenges related to collecting phone numbers during the mass distributions, and missing data from phone follow-up sessions limited the analysis. Additionally, response bias and self-reporting on the use of kit items may have resulted in over-estimating beneficiaries’ compliance with kit instructions. The number of recipients who were not reached by phone call (due to incorrect phone numbers or network coverage issues) was not recorded, thus limiting the representativeness of the answers. Finally, beneficiaries of the targeted distribution (non-health staff) were reached by phone interview after a longer delay than the other groups, which may have contributed to the higher rate of sickness/death in this group. Analysis was not weighted according to the length of time between distribution and interview, as the primary objective was to assess the use of kits and not the occurrence of disease.

The potential impact of distributing hygiene kits at community level in Liberia has been already acknowledged [12,16]. Merler et al. (2015) related decreasing trends of EVD occurrence with the increased isolation capacity, safe burials and household protection kits [17]. Atkins et al. (2016) alleged that the distribution in Monrovia started in “late October 2014” [16]. Nevertheless, this was the only mass distribution of hygiene kit occurring at the time and it started in week 36, 2014 (i.e. early September).

There is limited published knowledge on similar strategies of distributing household disinfection kit, and is not related to EVD. A study in Haiti addressed an intervention of targeted distribution of household disinfection kits to households of patients admitted to cholera treatment centres [6]. Authors highlighted the importance of integrating the distribution in a tailored HP communication strategy, as the means to increase the adequate usage of kits [18]. Drawing further parallels with cholera control measures is nonetheless risky, as the objective of the mass distribution in Monrovia was not one of ‘community case management for EVD’, but as rather an exceptional protection measure to halt the EVD transmission.

Our study suggests a number of recommendations to be considered in future EVD outbreaks: i) encouraging the use of chlorine for personal and household hygiene, even among households not experiencing sickness and/or death, hints at a possible relevance of a “chlorine-only mini-kit”; ii) “Full kit” distribution should be considered in situations of EVD outbreaks where prompt isolation cannot be offered through overwhelming caseloads. A distribution of hygiene kits could be considered under circumstances where security constraints limit transportation of the sick or dead or when disinfection teams cannot reach the affected households. Those incidents are observed during the persistent second largest EVD outbreak on record (2018–2019) in Eastern Democratic Republic of Congo (North Kivu, South Kivu, Ituri provinces). A mixed methods study showed that while social resistance to EVD control efforts was prevalent, the majority of respondents had compliant attitudes towards EVD control.; iii) consider more research into the possible impact of the kit distribution on EVD transmission in the community. Qualitative research would be helpful to assess whether the kit gives people a false sense of security, or identifying difficulties using the kits in order to better frame their expectations and needs.

### Conclusion

In the words of one of the MSF distribution teams, the household disinfection kits were “an imperfect solution for a difficult situation”. At the peak of an unprecedented outbreak of Ebola, the distribution of household disinfection kits was feasible and kits were appropriately used by the majority of the beneficiaries. In similar circumstances in the future, the intervention should be considered.

## Supporting information

S1 Protocol Study Protocol. Household disinfection kit in Monrovia.
(DOCX)

S1 Questionnaire (DOC)

S1 Spreadsheet Questionnaire variables and data. (XLSX)
