# Supplementary material for: Distribution of household disinfection kits during the 2014-2015 Ebola virus outbreak in Monrovia, Liberia: The MSF experience
Source: PLoS Negl Trop Dis. 2020 Sep 21;14(9):e0008539. doi: 10.1371/journal.pntd.0008539 (PMC7529189; doi:10.1371/journal.pntd.0008539)
Supplement: S1 Protocol — Household disinfection kit in Monrovia. (DOCX) [file pntd.0008539.s001.docx]

**Operational Research Study Protocol**

**Ebola kits distribution during the Ebola epidemic in Monrovia, Liberia: The MSF experience**

**Country:**  Liberia

**Site:**  Monrovia

**Investigators:**

Médecins Sans Frontières – Operational Centre Brussels, Monrovia: *Anna Halford, Saverio Bellizi*

Ministry of Health and social welfare– Liberia: *Luke Bawo, Moses Massaquoi*

Médecins Sans Frontières – Operational Centre Brussels: *Peter Maes*

Médecins Sans Frontières –Operational Research Unit, Luxembourg: *Engy Ali, Rafael Van de Bergh*

**Corresponding author:**

Dr. Engy Ali

Médecins Sans Frontières, Luxembourg

68 Rue de Gasperich, L-1617, Luxembourg.

Fax: (352) 335133 Tel : (352) 332522 e-mail : engy.y.ali@gmail.com

# Introduction

## The current Ebola outbreak is the first in West Africa and the largest in history – In August 2014, the WHO declared the outbreak as a public health emergency of international concern ^1^. Ebola virus is a filovirus, one of the most virulent human pathogens. The viral strain responsible for the current outbreak has been identified as the Zaire strain which is the most virulent strain associated with mortality rates as high as 90% ^2^. During outbreaks, transmission occurs through direct person-to-person contact or through contact with body fluids of infected EBV patients. The ritual washing of EBV victims at funerals and lack of infection control in health facilities can amplify an Ebola epidemic. ^3,4^

## EBV outbreaks were rare and usually occurred in rural areas with low population density and residents were rarely travelled far from home, these outbreaks were quickly contained. The current EBV epidemic in West Africa has spread widely and rapidly as result of the extensive movements of infected persons, the number of reported cases far exceeds the total number of cases in all previous outbreaks combined. ^5, 6^

Liberia is the most affected country in West Africa and the numbers of deaths from Ebola started to spiral. Over all by the 10th of October, the WHO declared 8376 (probable, confirmed and suspected) cases and 4024 deaths from EVD- of 4076 cases and 2316 death were reported in Liberia. ^5^

In August 2014, Medecins Sans Frontieres (MSF) has responded to the Ebola outbreak in Liberia. The MSF response included the establishment of an Ebola treatment (ETU) with a capacity of 240 beds in Monrovia. However, in such unique urban context of an Ebola outbreak, the conventional control measures were not enough to defeat the disease. In an attempt to slow down the epidemic, MSF decided to distribute Ebola protective kits in the zones most affected by EBV in Monrovia and among those who were at a high risk of contamination e.g health workers, relatives of admitted patients. The kits contained a bucket, chlorine, surgical gown, mask, gloves, and goggles. The objective of the kit is to be used to care for sick persons or to handle any dead body while waiting for the ambulance. The distribution was accompanied by health promotion (HP) activities in the community. In order to ensure the optimal and correct use of the kits, the HP activities were reinforced by follow-up phone calls to the recipients, to enquire about the use of the kit and its correctness and also to deliver HP messages.

## Distribution of Ebola protective kits in such urban context is a novel intervention for MSF which involved considerable logistics and financial resources and it is thus vital to document the experience of distributing the kits and if they were well used by the community. There is lack of knowledge about such unconventional interventions which might help to control an Ebola outbreak. Therefore, in the urban context of Monrovia, we propose a study to document the intervention of distributing Ebola protective kits and their use by the recipients. This information would be useful to guide future similar interventions by MSF and other humanitarian actors.

**Operational research questions:**

In the context of Ebola outbreak in Monrovia, Liberia where protective kits were distributed:

The specific operational research questions are:

- Were these kits used for the intended purpose(s)?
  - If not, what they were used for?
  - If yes, were the kit materials used correctly?
  - Were the kit materials useful?

# Objectives of the study

Based on our experience of distributing protective kits during the Ebola outbreak in Monrovia, Liberia, the objectives of this study are to:

1. describe the contents and the distribution process of the Ebola protective kits with lessons learnt and challenges
2. assess whether the distributed kits were used correctly and for the intended purpose

# Methods

## Design

This is a retrospective study using routinely collected program data

## Study period and population

The study will include those who received the Ebola protective kits and were contacted by phone between September and November 2014.

## Study setting

Liberia is a poor country, positioned 175^th^ out of 187 countries on the human development index. ^7^ The estimated population in 2012 was 4 4,190,000 inhabitants. Liberia is divided into fifteen counties, which are subdivided into a total of 90 districts and further subdivided into clans. Montserrado is the smallest and the most populous county with 1,144,806 residents as of the 2008 census.^8^ After decades of civil war in Liberia which ended in 2003, almost 95% of the health care facilities were damaged. In 2008, Liberia had only 1 doctor and 27 nurses per 100,000 people. ^9^ The country is one with the worst health indicators in the world. The estimated under-5 mortality rate is 110 per 1000 live births and 994 maternal deaths per 100.000 live births. ^10^

Monrovia is the capital city located in Montserrado County. It is the country’s most populous city with a population of 1, 2 million, accounting for 29% of the total population of Liberia. Monrovia is divided into administrative district which are consequently divided into communities.

## Ebola protective kit distribution

- The distribution process included a total of 70,000 Ebola protective kits, distributed between September and November 2014. The details of the kit contents is shown in Box 1
- **Types of kit distribution:** there were two types of distribution;

1. Targeted distribution: to those who were at high risk of contamination which includes:

- All health workers in Greater Monrovia.
- Contact (relatives/accompanied person (s)) of the EBV patients admitted to MSF ETU
- Patients who were turned away from the MSF ETU during the peak period due to lack of admission beds
- Relatives of suspected patients in the community transferred by ambulance dispatch team and for those who were not transferred due to lack of admission beds in the ETUs.

1. Mass distribution: among the general population residing in three urban slum zones of Monrovia namely; Clara Town, New Kru Town, and West Point. These zones are the most populous in Monrovia, with the lowest socio-economic status and were mostly affected by the Ebola outbreak.

- **Process of distribution:**

1. Targeted distribution: This was done directly to patients at the MSF ETU and indirectly through the MOH ambulance team to suspects in the community. Distribution to health workers was also done through the MOH. During the distribution, phone numbers of all recipients were reported and listed.
2. Mass distribution: This was done directly in the community. With the support of the community leaders and chairmen in the communities, the number of households was identified. The distribution included two kits for each household. Due to the logistic difficulty of such distribution, 2% of the phone numbers of the recipients were randomly collected.

- **Health promotion:**
- Health promotion (HP) sessions and demonstration on the correct use of the kits targeted all recipients. These sessions were held in the area of distribution mainly in the community and hospitals.
- An MSF call center was established with four trained health promoters who used call the recipients of the kits in order to confirm the correct use of the kits and provide HP messages.
- During the phone call, all recipients were explained the purpose of the phone call and verbal permission was sought to pursue with the questionnaire. A pretested semi-structure questionnaire was used to gather information on the basic demographic information of the recipient’s household and to assess the correct use of the different items of the kits. The questionnaire is shown in Annex 1.
- The phone calls were targeted 3-5 days after distribution; this was followed by a second follow-up call 10-15 days after distribution. The same questionnaire was used for both calls.

*Box1: Contents of the Ebola protective kit.*

| **ITEMS** | **QUANTITY** |
| --- | --- |
| [CHLORINE, NaDCC granules,](https://tukul.msf.org/policies-guidelines/itc-international-technical-coordination/itc-catalogues/decompressed/eng/catalog/logistic_folder/CWAT/CWATYCHN1--_eng.html)(with dosing spoon) | 0,5 kg |
| DISPOSABLE LATIX GLOVES | 100 |
| REUSABLE RUBBER GLOVES FOR CLEANING | 4 |
| PLASTIC BUCKET WITH LID (20 L) | 1 |
| PLASTIC BUCKET WITH TAP (20 L) | 1 |
| PLASTIC HAND SPRAYER (1L) | household type |
| SOAP (100g) | 5 |
| PLASTIC BAG (100L) | 20 bags per roll |
| DISPOSABLE SURGICAL [GOWN](https://tukul.msf.org/policies-guidelines/itc-international-technical-coordination/itc-catalogues/decompressed/eng/catalog/volume2/ELIN/ELINGOWSS_eng.html) | 4 |
| DISPOSABLE SURGICAL MASK | 25 |
| BASIC GOGGLES | 1 |
| LEAFLET N HOW TO USE THE KIT | 1 |

**Study sample**

To identify an assumed 50% correct use of the kit by its recipients, with a 95% confidence and 5% precision, a total of 383 recipients for all types of distribution groups will be randomly selected from the database of the call centre.

## Data source and Statistical analysis

The study will include the data of the semi- structured questionnaire used during the phone calls and the project’s routinely collected data of the total number of distributed kits and areas of distribution. Data of the questionnaire is entered into Excel spread sheet. Data analysis will be done using EpiData software version 2.2 (EpiData Association, Odense, Denmark). Frequencies (%) will be calculated to describe the use of the kits by the community and comparison between types of distribution per population.

# Ethical issues

*Consent and data confidentiality*

- The study will involve a retrospective analysis of routinely collected program data and verbal agreement was sought from all recipients contacted by phone to answer the questions.
- All data are held in strict confidentiality as is normal practice. No patient names or identifying information will be used in any presentation or publication.

*Specific patient benefits or harms:*

There are no specific patient benefits or harms associated with this study, as it involves retrospective analysis of routinely collected programmatic data. Data collection didn’t involve contacts with patients.

*Community participation and benefits*

- The community leaders and chairmen are informed and supporting the distribution process. They will be also informed about the study and its results.

Feedback and dissemination of results

- The results of this study and their potential implications will be made known to MSF projects, the operations department, health workers and partners involved with the Ebola emergency response.
- National and international platforms will be used to share knowledge
- In order to disseminate the information gained, attempts will be made to publish the experience in an international open-access peer-reviewed journal.

Implication for policy and practice

- There is currently very limited literature on the unconventional methods to control Ebola epidemic in an urban context. The lessons learnt relevant for MSF, and relevant for other actors would guide planning the on-going and future Ebola control programs.

Collaborative partnership

- The study will involve partners from the Ministry of Health of Liberia, and the MSF Operational centre in Brussels.
- All partners will be represented on the study manuscript and any eventual publication.

*Ethics review*

- The MSF project in the Liberia is formally approved by the Ministry of Health.
- The study doesn’t include any confidential data of patients.
- Ethical approval will be sought from the Ethical Review Boards of MSF, Geneva, Switzerland.

*Funding*

The study will not require any specific funding as the staff involved in the project are full time MSF employees. The project is funded by MSF-Operational Centre Brussels. Costs of open access publication will be covered by LUXOR.

# References

1. World Health Organization. WHO Statement on the Meeting of the International Health Regulations Emergency Committee Regarding the 2014 Ebola Outbreak in West Africa. 2014
2. Baize et Al. Emergence of Zaire Ebola Virus Disease in Guinea — Preliminary Report. The new England Journal of Medicine. 2014; DOI: 10.1056/NEJMoa1404505.
3. Feldmann et All. Ebola haemorrhagic fever.*Lancet*. 2011; 377(9768): 849–862.
4. MacNeil at Al. Ebola and Marburg Hemorrhagic Fevers: Neglected Tropical Diseases? *Plos One Neglecte Tropical Diseases.* 2012; e1546.
5. World Health Organization: Ebola response roadmap situation report: 10 October 2014. http://apps.who.int/iris/bitstream/10665/136020/1/roadmapsitrep_10Oct2014_eng.pdf?ua=1 (Accessed on October 11, 2014).
6. World Health Organization. Ebola situation in Liberia: non-conventional interventions needed. http://www.who.int/mediacentre/news/ebola/8-september-2014/en/# (Accessed on September 08, 2014).
7. UNDP Human Development Report 2013
8. World Health Organization.World Health Statistics 2014.*World Health Organization.*2014.
9. ["Liberia: Breathing Life into ailing healthcare system"](http://www.irinnews.org/report.aspx?reportid=60788). Integrated Regional Information Networks. UN Office for the Coordination of Humanitarian Affairs.
10. UNICEF: Liberia Basic Indicators 2014.
