# Supplementary material for: Distribution of household disinfection kits during the 2014-2015 Ebola virus outbreak in Monrovia, Liberia: The MSF experience
Source: PLoS Negl Trop Dis. 2020 Sep 21;14(9):e0008539. doi: 10.1371/journal.pntd.0008539 (PMC7529189; doi:10.1371/journal.pntd.0008539)
Supplement: S1 Questionnaire — (DOC) [file pntd.0008539.s002.doc]

**Questionnaire for follow up by phone**

Date of the call: Contact list:

Name: Surname: Telephone N:

Zone: Community: Date kit distributed:

***General questions***

1. How many people do normally live in your house? _________
2. How many people are living now in your house? ___________
3. How many families in your household? ___________________
4. Has there been any sick person with fever and/or vomit and/or diarrhea in your household since you got the kit? Yes□ No □
5. If yes, how many?________ What is her/his job?_____________
6. Has she/he been hospitalized? Yes□ No □
7. Has anybody died in your household since you got the kit? Yes□ No □ Job?
8. Has anybody died from your household since the outbreak? Yes □ No □ Job?_________

***Specific questions***

1. Are you using the kit? Yes□ No □
2. If yes, then specify the purpose?_______________
3. Was the kit complete? Yes□ No □
4. Did you get training on the use of the kit? Yes□ No □
5. Were the instructions understandable? Yes□ No □
6. Was the leaflet understandable? Yes□ No □
7. Do you use chlorine to wash your hands? Yes□ No □
8. If yes, how many spoons of chlorine powder by bucket of water? Correct□ Not correct□
9. Do you use chlorine to clean? Yes□ No □
10. If yes, how many spoons of chlorine powder by bucket of water? Correct□ Not correct□
11. **Only if death occurred (*look at general questions*!):** did you use chlorine to wash the dead body?

Yes□ No□ Done by burial team□

1. **Only if death occurred (*look at general questions*!):** how many spoons of chlorine powder by bucket of water for the chlorine used to wash dead body? Correct□ not correct□
2. If you have dirty mattress/clothes of a sick person, do you spray it? Yes□ no□
3. **Only if death occurred (*look at general questions*!):** did you dispose mattress/clothes of the dead person? Yes□ no□ room/house locked□
4. Are you using gloves? Yes□ no□
5. Are you washing disposable gloves with chlorine after each contact and before taking them off? Yes□ no□
6. Do you use thick gloves when manipulating body fluids or material contaminated by body fluids? Yes□ no□
7. Are you using the hand sprayer when disinfecting surfaces? Yes□ no□
8. Do you disinfect the outside of plastic bags before disposing contaminated material? Yes□ no□
9. Do you place the bag with contaminated material in a second bag? Yes□ no□
10. How do you manage bags with contaminated material? Burn□ Bury□ other□
11. Have you run out of any of the kit items? Yes□ no□ *specify
12. Are you using the surgical gown when manipulating a sick person or body fluid? Yes□ no□
13. **Only if death occurred (*look at general questions*!):** did you use the surgical gown when manipulating a dead person? Yes□ no□
14. Are you using the mask when manipulating a sick person or body fluid? Yes□ no□
15. **Only if death occurred (*look at general questions*!):** did you use the mask when manipulating a dead person? Yes□ no□
16. Did you dispose you disposable gloves once you took them off? Yes□ no□
17. Have you had any problem with the kit? Yes□ no□
18. If yes, then specify the problem?____________________________
19. Do you think the kits materials were useful? Yes□ no □
